# Efficacy of transarterial chemoembolization monotherapy or combination conversion therapy in unresectable hepatocellular carcinoma: A systematic review and meta-analysis

**DOI:** 10.3389/fonc.2022.930868

**Published:** 2022-08-01

**Authors:** Weiwei Li, Yinxuan Pei, Zixiang Wang, Jinlong Liu

**Affiliations:** Department of Hepatobiliary Surgery, Affiliated Hospital of Chengde Medical University, Chengde, China

**Keywords:** conversion therapy, transarterial chemoembolization (TACE), unresectable, liver cancer, surgery

## Abstract

**Background:**

Hepatocellular carcinoma (HCC) is a highly malignant disease with poor prognosis, and most cases were already considered unresectable at the time of presentation. Conversion therapy, as an emerging treatment, is designed to provide patients with initially unresectable hepatocellular carcinoma (uHCC) the opportunity to undergo radical resection. At present, conversion therapy for patients with uHCC remains controversial. Transarterial chemoembolization (TACE) is currently the most widely selected treatment for uHCC, but its efficacy as a conversion therapy remains controversial.

**Methods:**

We compared and evaluated the conversion rate for and tumor response to TACE monotherapy or combination therapy. Meanwhile, postoperative complications and overall survival (OS) in uHCC patients who underwent conversion therapy were also analyzed.

**Results:**

A total of 18 studies were included in this meta-analysis. The conversion rate for triple therapy [TACE in combination with tyrosine kinase inhibitors (TKIs) and immune checkpoint inhibitors (ICIs)] was 42% [95% confidence interval (CI), 0.29–0.56], higher than any other group [TACE monotherapy: 10% (95% CI, 0.08–0.12), bigeminy therapy: 19% (95% CI, 0.06–0.36)]. Meanwhile, triple therapy yielded a better tumor response than TACE monotherapy or bigeminy therapy. Among the patients with successful surgical resection after conversion therapy, the pooled postoperative OS rates at 1, 2, and 5 years were 90% (95% CI, 0.81–0.97), 58% (95% CI, 0.42–0.73), and 42% (95% CI, 0.26–0.60), respectively, and the major postoperative complications were biliary leakage (7%; 95% CI, 0.03–0.12) and liver failure (3%; 95% CI, 0.00–0.07).

**Conclusion:**

TACE conversion therapies showed good conversion rates, especially the triple therapy of TACE in combination with TKIs and ICIs. Surgical resection after successful conversion therapy could maximize the outcome of patients with uHCC.

## Introduction

Hepatocellular carcinoma (HCC) is the sixth most common cancer and ranks third in terms of the mortality rate worldwide (1, 2). The preferred treatment for HCC is surgery, which is the only way to achieve long-term survival or even a cure. However, most cases are considered unresectable at the time of presentation, and treatment options are limited, with very poor prognoses. In recent years, conversion therapy for unresectable hepatocellular carcinoma (uHCC) has attracted increased interest. Conversion therapy is designed to achieve tumor downstaging and provide patients with initially unresectable or borderline resectable malignancies the opportunity to undergo radical resection (3). This approach is now the established treatment strategy for many solid tumors, including colorectal cancer (4). However, it is still controversial in uHCC and has not been promoted in practice.

Transarterial chemoembolization (TACE) is the most widely selected treatment for uHCC and is considered the first-line treatment for patients with intermediate or advanced HCC. However, TACE monotherapy does not seem ideal for conversion therapy (5, 6). In addition to TACE, other common local treatments include hepatic arterial infusion chemotherapy (HAIC) and radiotherapy (RT) (7). Common systemic treatments include chemotherapy, targeted therapy, and immunotherapy (8). With the popularity of other treatments, such as local or systemic treatments, combination therapy seems to be the trend, which may provide cumulative benefits in efficacy beyond what either approach alone provides (3). Effective conversion therapy strategies are still being explored. Conversion therapy may be a viable option to improve patient outcomes, but the exact effect is still unclear and controversial (9). Based on the discussion of the efficacy of TACE conversion therapy for uHCC, we analyzed the conversion rate, tumor response, safety, and prognosis in a meta-analysis.

## Materials and methods

### Search strategy

We searched for relevant articles in the PubMed, Embase, and Web of Science literature databases from 1 January 2010 to 20 June 2022 without language restrictions. The detailed search strategy was as follows: “[(hepatocellular carcinoma) OR (hepatocellular cancer) OR (liver cancer) OR (hepatic neoplasm) OR (HCC)] AND [(TACE) OR (c-TACE) OR (DEB-TACE) OR (transcatheter arterial chemoembolization) OR (transarterial chemoembolization)] AND [(downstaging) OR (downstage) OR (conversion therapy) OR (preoperative treatment) OR (preoperative therapy)].” References from the included articles and relevant published reports were also hand-searched.

### Inclusion and exclusion criteria

The processes of recognition, inclusion, and exclusion of articles were conducted in accordance with the Preferred Reporting Items for Systematic reviews and Meta-Analyses guidelines. In this meta-analysis, eligible articles were selected according to the following inclusion criteria: 1) patients with primarily uHCC and those with the extrahepatic disease for which final resection was not possible or extensive local disease; 2) intervention with any TACE treatment that reduces tumor load and tumor stage (including TACE monotherapy or combination therapy); and 3) evaluation of the success rate of conversion therapy at minimum as a study result. There were no restrictions on study design, language, or publication status. The following articles were excluded: 1) duplicate articles, reviews, case reports, comments, and editorials; 2) those where subjects were a mix of other cancer patients and data could not be presented separately; 3) those where <5 patients were enrolled; and 4) the study without ethics approval and the informed consent of patients. If several qualified papers were from the same author (using the same case series), the study with the most detailed or up-to-date information was selected for inclusion in the present research.

### Data extraction and quality assessment

Two authors independently extracted data from the included articles and summarized the final results. Data extracted from the eligible studies included study characteristics (first author, publication year, study design), basic patients’ information (number, age, gender, follow-up time, etc.), conversion therapy modality, conversion rate, tumor response, postoperative complications, and prognosis. Each author also independently assessed the quality of the included studies. The Institute of Health Economics Quality Appraisal (IHEQA) checklist (10) was used for quality assessment. Any disagreements were resolved through discussion.

### Statistical analysis

We calculated event rates for outcomes (conversion rate, tumor response, postoperative complications, and prognosis) and estimated 95% confidence intervals (CIs). We used Cochrane’s Q statistic and the I^2^ statistic to determine heterogeneity between eligible studies. Significant heterogeneity was defined as I^2^ >50% or a Q-test with P < 0.10. A random-effects model was used when significant heterogeneity was observed; otherwise, we chose a fixed-effects model. Publication bias was assessed using the funnel plot with Egger’s test, where a P-value <0.05 indicated significant publication bias. Meta-regression was also conducted to explore and explain diversity among the results of different studies when significant heterogeneity was observed. This meta-analysis was performed using the R version 4.1.2 (R Foundation for Statistical Computing, Vienna, Austria) and adhered to the Preferred Reporting Items for Systematic reviews and Meta-Analyses guidelines.

## Results

### Literature search results

**Figure 1:**
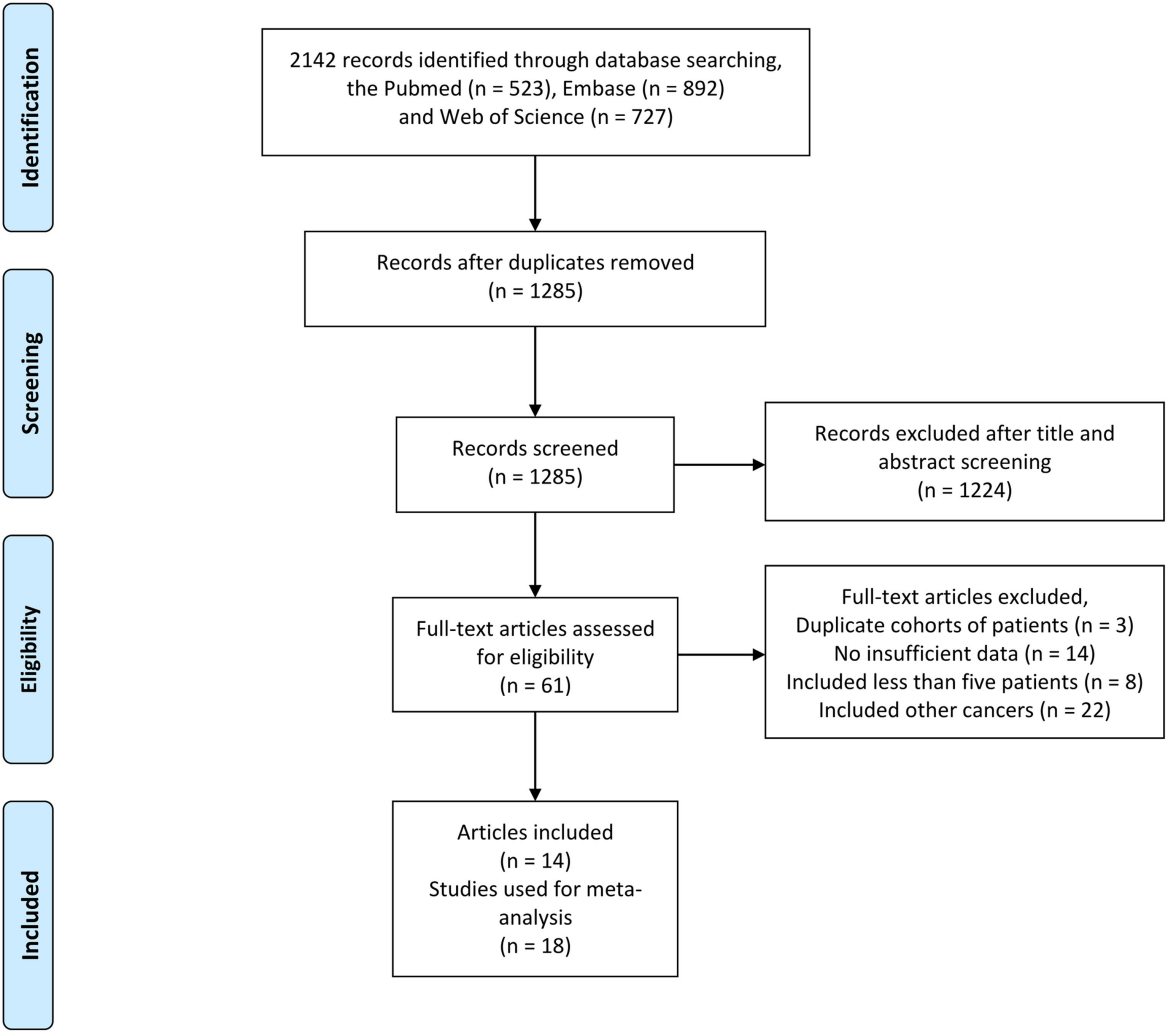
Flowchart outlining the search strategy and details on the studies finally included in the meta-analysis.

We found a total of 2,142 articles using the above search method in PubMed, Embase, and the Web of Science. After a review of all titles and abstracts, 2,081 articles were excluded because they were not relevant to the current analysis. The remaining 61 potentially relevant articles were screened by full-text review, and 47 were excluded for the following reasons: reported duplicate cohorts of patients (n = 3 articles), insufficient data (n = 14 articles), included <5 patients (n = 8 articles), and included other cancers (n = 22 articles). Finally, 14 articles reporting 18 studies were included in our meta-analysis. The screening process for eligible studies is presented in **Figure 1**.

### Characteristics and quality assessments of the selected studies

A total of 14 articles (11–24) presenting the details of 18 studies were included in our meta-analysis. There were 2,528 patients undergoing conversion therapy and 331 patients undergoing surgical resection after successful conversion therapy. Conversion therapies included TACE monotherapy [nine studies (15, 17, 18, 21–24)] or TACE combined with other therapies [nine studies (11–16, 19, 20)]. All patients were diagnosed with HCC by pathology or non-invasive methods (imaging, serology, etc.). The criteria for unresectable patients included varied from study to study. Studies on TACE monotherapy tended to focus on unresectable patients whose liver cancer was too bulky for resection, with inadequate surgical margins or located centrally at the hepatic hilus, and exclude patients with vascular invasion or distant metastasis. By contrast, studies on TACE combination therapy, especially in combination with systemic therapy, included patients with diffuse liver tumors, major vascular invasion, or extrahepatic metastasis. All studies mentioned liver function, and the included patients had a normal liver function or compensatory cirrhosis (Child–Pugh score A or B). Some articles (11, 13, 14, 16, 18, 20, 21, 23) also evaluated the Eastern Cooperative Oncology Group performance status (ECOG PS) of the enrolled patients. The main characteristics of the studies are summarized in **Table 1**. We also conducted a quality assessment of the included studies. All studies were considered to be of acceptable quality according to the IHEQA checklist.

**Table 1:** Characteristics of studies selected for analysis.

First author	Year	Study design	Number of patients	SexM/F	Age years	Intervention	Types of intervention	Tumor grade andmain features	Patient condition	Study period	Mean Follow up month
Su (12)	2022	Prospective, single-center	30	NA	NA	TACE+RT	Bigeminy therapy	NA (macroscopic vascular invasion)	NA	03.2018–05.2020	NA
Song (13)	2022	Retrospective, single-center	37	NA	NA	TACE+TKI+ICI	Triple therapy	NA (vascular invasion, extrahepatic metastasis)	Child–Pugh A/B, ECOG PS 0–1	12.2018–10.2020	NA
Zhang (11)	2022	Prospective, multicenter	38	35/3	NA	TACE+TKI+ICI	Triple therapy	BCLC B/C	Child–Pugh A/B, ECOG PS 0–1	09.2020–05.2021	8.33
Chen (1) (16)	2021	Retrospective, multicenter	70	37/33	58 (36–69)	TACE+TKI+ICI	Triple therapy	BCLC B/C (no bile duct invasion, PVTT or symptomatic brain metastases)	Child–Pugh A, ECOG PS 0–1	07.2016–07.2020	27
Chen (2) (16)	2021	Retrospective, multicenter	72	38/34	57 (35–68)	TACE+TKI	Bigeminy therapy	BCLC B/C (no bile duct invasion, PVTT or symptomatic brain metastases)	Child–Pugh A, ECOG PS 0–1	07.2016–07.2020	27
Li (1) (15)	2021	Retrospective, single-center	42	37/5	NA	cTACE	Monotherapy	BCLC A/B (no vascular invasion or extrahepatic metastasis)	Child–Pugh A	01.2015–07.2019	47.8
Li (2) (15)	2021	Retrospective, single-center	41	30/11	NA	TACE+HAIC	Bigeminy therapy	BCLC A/B (no vascular invasion or extrahepatic metastasis)	Child–Pugh A	01.2015–07.2019	19.6
Cai (17)	2021	Prospective, single-center	32	20/12	60.0 ± 10.8	DEB-TACE	Monotherapy	BCLC B (no vascular invasion, bile duct invasion or extrahepatic metastasis)	Child–Pugh A/B	05.2016–03.2017	35.4
Wu (14)	2021	Retrospective, multicenter	62	56/6	57 (23–75)	TACE+TKI+ICI	Triple therapy	BCLC A/B/C (vascular invasion, extrahepatic metastasis)	Child–Pugh A, ECOG PS 0–1	11.2018–12.2020	12.2
Chiu (1) (18)	2020	Retrospective, single-center	19	18/1	63.7 (27.0–86.0)	cTACE	Monotherapy	BCLC A/B/C (no extrahepatic metastases or PVTT)	Child–Pugh A/B, ECOG PS 0–1	01.2016–03.2019	12
Chiu (2) (18)	2020	Retrospective, single-center	42	32/10	67.4 (41.0–87.6)	DEB-TACE	Monotherapy	BCLC A/B/C (no extrahepatic metastases or PVTT)	Child–Pugh A/B, ECOG PS 0–1	01.2016–03.2019	12
Song (19)	2019	Retrospective, single-center	652	NA	NA	TACE+RT	Bigeminy therapy	NA (macroscopic vascular invasion)	NA	01.2010–02.2016	38
Yoon (20)	2018	Prospective, single-center	45	38/7	55 (42–77)	TACE+RT	Bigeminy therapy	BCLC A (vascular invasion or bile duct invasion; no extrahepatic metastasis)	Child–Pugh A, ECOG PS 0–1	07.2013–10.2016	7.8
Wu (1) (21)	2018	Retrospective, single-center	30	27/3	52.83 ± 6.13	cTACE	Monotherapy	BCLC B/C (no vascular invasion or extrahepatic metastasis)	Child–Pugh A/B, ECOG PS 0–2	06.2016–02.2017	6
Wu (2) (21)	2018	Retrospective, single-center	24	22/2	56.25 ± 7.47	DEB-TACE	Monotherapy	BCLC B/C (no vascular invasion or extrahepatic metastasis)	Child–Pugh A/B, ECOG PS 0–2	06.2016–02.2017	6
He (22)	2017	prospective, single-center	41	37/4	NA	cTACE	Monotherapy	BCLC A/B (no vascular invasion or extrahepatic metastasis)	Child–Pugh A	10.2015–10.2016	NA
Zhang (23)	2016	Retrospective, single-center	831	NA	NA	cTACE	Monotherapy	NA (no extrahepatic metastasis or PVTT)	Child–Pugh A/B, ECOG PS 0–2	06.2004–12.2014	42.2
Shi (24)	2012	Prospective, single-center	420	NA	NA	cTACE	Monotherapy	NA (too bulky for resection or situated centrally at the hepatic hilus, macroscopic vascular invasion)	Child–Pugh A/B	01.2004–12.2008	48

TACE, transarterial chemoembolization; cTACE, conventional transarterial chemoembolization; DEB-TACE, drug-eluting beads transarterial chemoembolization; RT, radiotherapy; HAIC, hepatic arterial infusion chemotherapy; TKI, tyrosine kinase inhibitor; ICI, immune checkpoint inhibitor; BCLC, Barcelona Clinic Liver Cancer; PVTT, portal vein tumor thrombus; ECOG PS, Eastern Cooperative Oncology Group performance status; NA, not available; M, male; F, female.

### Conversion rate

**Figure 2:**
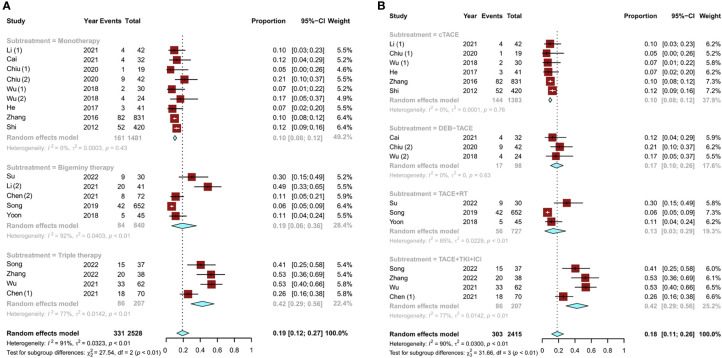
Pooled analysis of the conversion rate of uHCC: **(A)** TACE monotherapy and TACE combination therapy (bigeminy therapy or triple therapy) and **(B)** subgroup analysis of different combination therapies (cTACE, DEB-TACE, TACE combined with RT, and TACE in combination with TKIs and ICIs). uHCC, unresectable hepatocellular carcinoma; cTACE, conventional transarterial chemoembolization; DEB-TACE, drug-eluting beads transarterial chemoembolization; RT, radiotherapy; TKIs, tyrosine kinase inhibitors; ICIs, immune checkpoint inhibitors.

Among the nine studies (15, 17, 18, 21–24) on TACE monotherapy, six (15, 18, 21–24) used conventional TACE (cTACE) conversion therapy and three (17, 18, 21) used drug-eluting bead TACE (DEB-TACE) conversion therapy. Of the remaining nine studies (11–16, 19, 20), five (12, 15, 16, 19, 20) reported bigeminy therapy and four (11, 13, 14, 16) reported triple therapy. Among the bigeminy therapy studies, there were three studies (12, 19, 20) on TACE combined with RT, one study (15) on TACE combined with HAIC, and one study (16) on TACE combined with tyrosine kinase inhibitor (TKI) therapy. The remaining four triple therapy studies (11, 13, 14, 16) covered TACE in combination with TKI and immune checkpoint inhibitor (ICI) therapy. Conversion rates for TACE monotherapy ranged from 5% (18) to 53% (11, 14). The overall pooled conversion rate for TACE monotherapy was 10% (95% CI, 0.08–0.12). Compared to TACE monotherapy, combination therapies had higher overall conversion rates. The pooled conversion rates were 19% (95% CI, 0.06–0.36) for bigeminy therapy and 42% (95% CI, 0.29–0.56) for triple therapy (**Figure 2A**).

**Figure 3:**
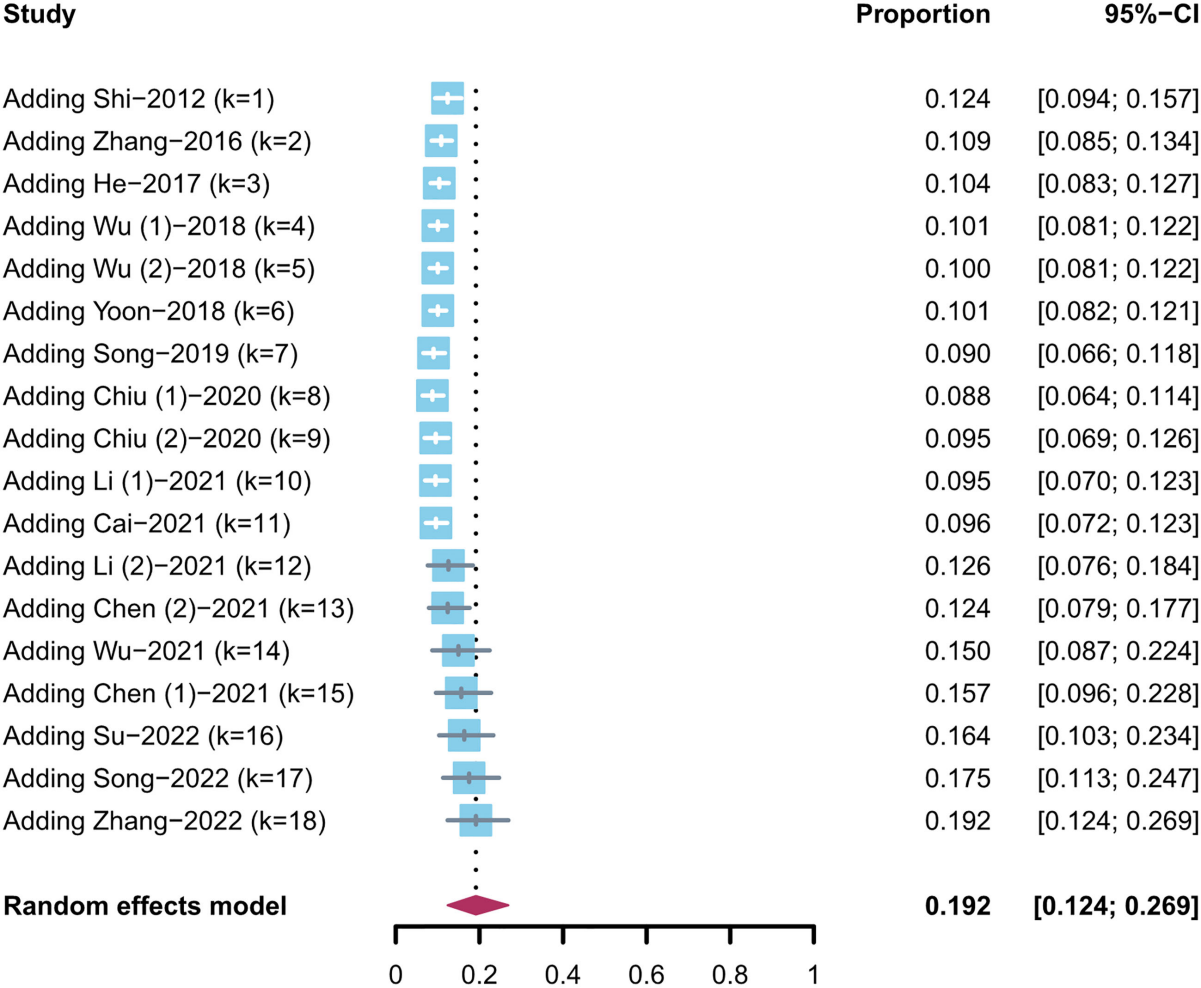
Cumulative meta-analysis of uHCC conversion rate (trend with time).

A subgroup analysis was also conducted for different combination therapies. In the two types of TACE monotherapy regimens, the pooled conversion rate of DEB-TACE was higher than that of cTACE [17% (95% CI, 0.10–0.26) vs. 10% (95% CI, 0.08–0.12)]. The conversion rate of TACE combined with RT was 13% (95% CI, 0.03–0.29). Notably, TACE in combination with TKI and ICI therapy had a higher conversion rate of 42% (95% CI, 0.29–0.56) than any other group (**Figure 2B**). Then, to examine how the evidence has changed over time, the cumulative meta-analysis was performed. The results did not deviate significantly (**Figure 3**).

### Tumor response

**Figure 4:**
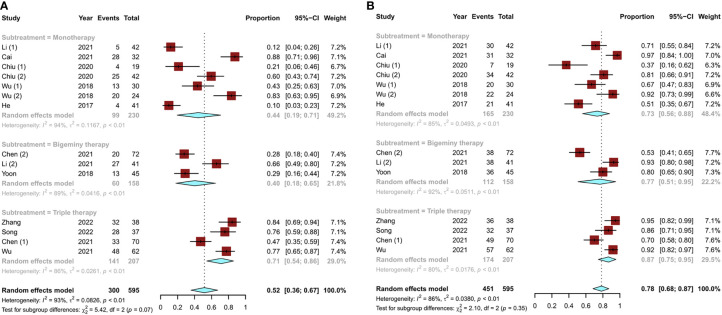
Tumor response to different TACE conversion therapies in uHCC: **(A)** objective response rate; **(B)** disease control rate.

A total of 14 studies (11, 13–18, 20–22) reported the modified Response Evaluation Criteria in Solid Tumors (mRECIST) for liver cancer. The objective response rate [ORR; defined as a complete response (CR) plus partial response (PR)] and disease control rate [DCR; defined as CR and PR plus stable disease (SD)] were used to assess tumor response. The pooled ORR of TACE monotherapy was 44% (95% CI, 0.19–0.71), and the overall ORRs of combination therapy were 40% (95% CI, 0.18–0.65) for bigeminy therapy and 71% (95% CI, 0.54–0.86) for triple therapy (**Figure 4A**). In terms of DCR, TACE monotherapy and TACE combination therapy (bigeminy therapy and triple therapy) increased successively but showed little difference (P = 0.35), accounting for 73% (95% CI, 0.56–0.88), 77% (95% CI, 0.51–0.95), and 87% (95% CI, 0.75–0.95), respectively (**Figure 4B**). Overall, triple therapy yielded a better tumor response than TACE monotherapy or bigeminy therapy.

### Safety and prognosis

**Figure 5:**
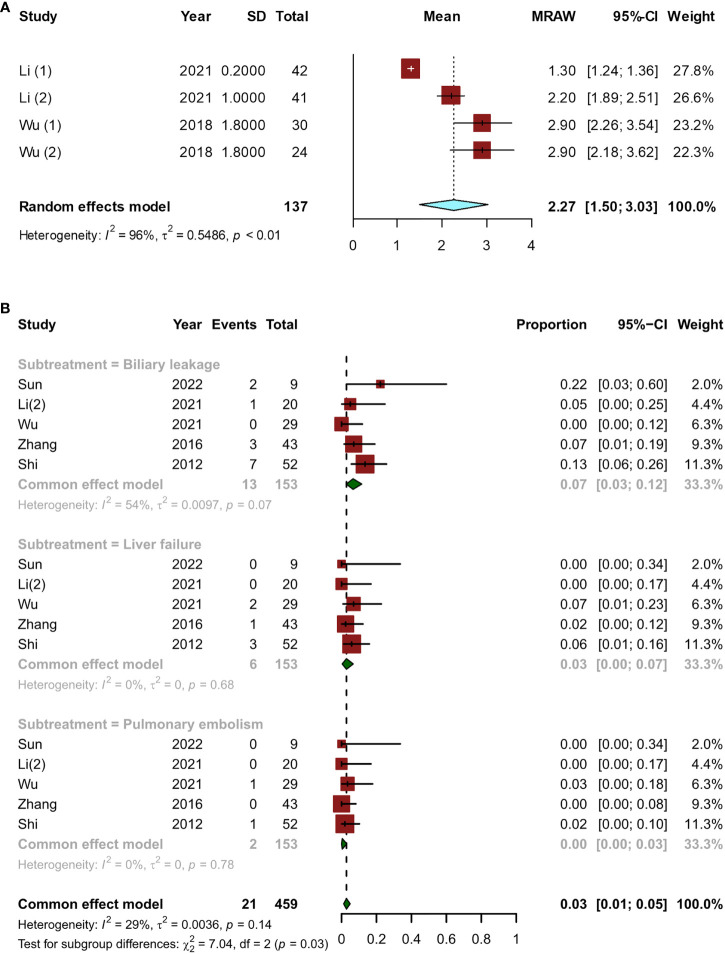
Short-term indicators in patients undergoing surgery after conversion therapy, including **(A)** the time between initial conversion therapy and resectable criteria; **(B)** major postoperative complications.

Most of the time, the period from the start of treatment to the confirmation of downstaging was within 6 months. The pooled overall downstage time was 2.27 (95% CI, 1.50–3.03) months (**Figure 5A**). A total of 331 patients who met the criteria for resection eventually underwent surgery, and there were five articles (12, 14, 15, 23, 24) with 153 patients that reported postoperative complications. The major postoperative complications were biliary leakage (7%; 95% CI, 0.03–0.12), liver failure (3%; 95% CI, 0.00–0.07), and pulmonary embolism (0%, 95% CI, 0.00–0.03) (**Figure 5B**).

**Figure 6:**
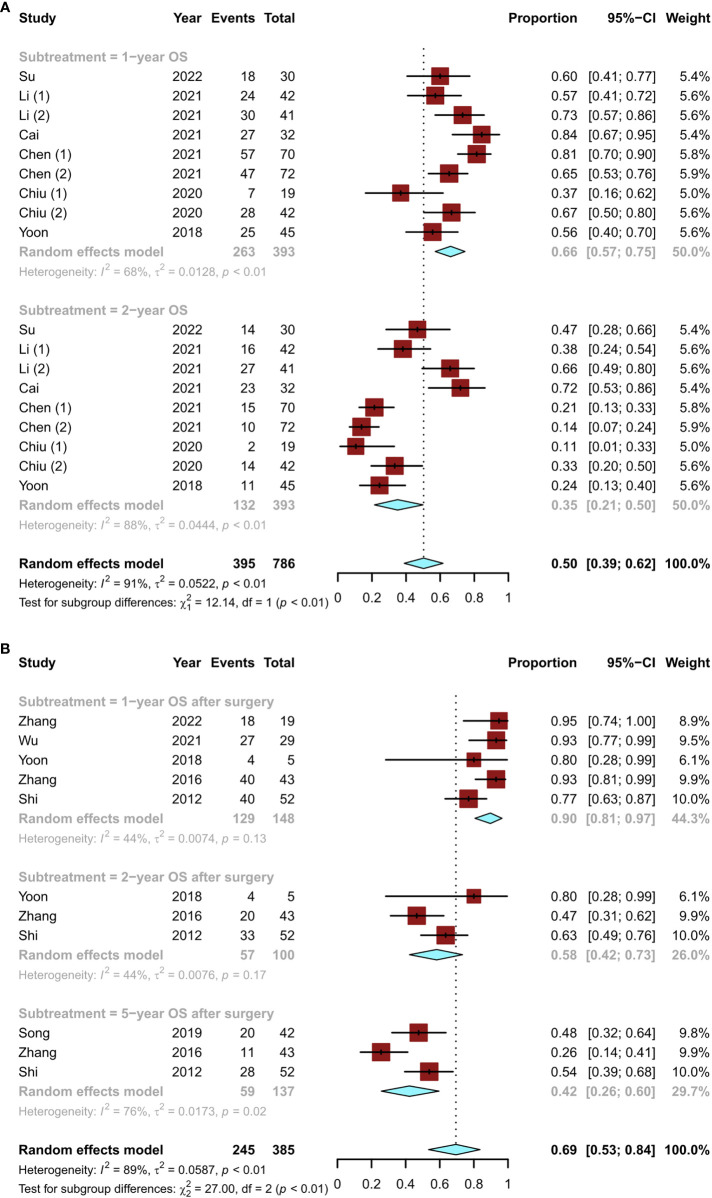
Details of the overall survival of patients who underwent conversion therapy, including the **(A)** overall survival of patients initially treated with conversion therapy and **(B)** postoperative overall survival in patients who underwent successful surgical resection after conversion therapy.

In terms of long-term survival outcomes, nine studies (12, 15–18, 20) revealed the overall survival (OS) of patients initially treated with conversion therapy, with 1- and 2-year OS rates of 66% (95% CI, 0.57–0.75) and 35% (95% CI, 0.21–0.50), respectively (**Figure 6A**). A total of six studies (11, 14, 19, 20, 23, 24) described the postoperative OS in patients who underwent successful surgical resection after conversion therapy. The lowest 1-year postoperative OS rate was 77% and the highest was 95%. The pooled OS rates at 1, 2, and 5 years were 90% (95% CI, 0.81–0.97), 58% (95% CI, 0.42–0.73), and 42% (95% CI, 0.26–0.60), respectively (**Figure 6B**). Thus, the OS of patients undergoing surgical resection after conversion therapy was significantly increased.

### Meta-regression and publication bias analysis

Meta-regression was performed to explore the origin of heterogeneity on conversion rate. However, owing to the limited number of studies included, the differences in age, sex, and study design did not have a statistically significant effect on heterogeneity. The result of meta-regression indicated that the origins of heterogeneity were relatively complex (**Supplementary Figure S1**).

The funnel plots with Egger’s tests were performed to assess the effect of publication bias in this meta-analysis. No significant publication bias existed based on an analysis of the conversion rate of TACE monotherapy (P = 0.4716), bigeminy therapy (P = 0.0866), and triple therapy (P = 0.5489) groups (**Supplementary Figure S2**).

## Discussion

The treatment of HCC has progressed rapidly in the past few years, and patient outcomes have improved significantly over time. However, the removal of HCC remains the only strategy likely to lead to an eventual cure. Conversion therapy, as an emerging treatment for uHCC in the middle and late stages of the disease, aims to reduce the tumor burden through downstaging, enabling patients to undergo surgical resection (25, 26). However, conversion therapy for patients with uHCC remains controversial (27). Currently, TACE has been widely recognized as an effective treatment option for uHCC. cTACE is usually carried out by combining lipiodol with chemotherapy drugs to block the tumor’s supplying arteries and increase the concentration of chemotherapy drugs in tumor tissue, leading to tumor infarction and necrosis (26). Unfortunately, the conversion rate for cTACE monotherapy does not seem to be ideal, being only 10%. First introduced in 2006 (28), DEB-TACE slowly releases chemotherapeutic drugs, thereby maintaining longer drug concentrations. Compared to cTACE, DEB-TACE had shown higher conversion rates of 17% in current conversion therapy. Even so, the efficacy of TACE monotherapy in uHCC conversion therapy remains limited, with an overall conversion rate of only 10% in our analysis. Other forms of treatment are urgently needed.

HAIC and RT, both topical therapies, are also used in the treatment of uHCC. Similar to TACE, both HAIC and RT monotherapies are not ideal. Studies have reported conversion rates as low as 11% for HAIC monotherapy (29) and <5% for RT monotherapy (30). Li et al. (15) reported that TACE combined with HAIC achieved a significantly higher conversion rate compared to cTACE (48% vs. 9%, P < 0.001) and was more advantageous in progression-free survival (PFS) (hazard ratio, 0.38; 95% CI, 0.20–0.86; P = 0.003). TACE in combination with RT also showed better results than monotherapy, especially in patients with uHCC with macroscopic vascular invasion (31, 32). Similar to previous studies, the conversion rate of TACE combined with RT in this meta-analysis reached 13%. Even though the combination of local treatments does not seem to be satisfactory, combination therapy seems to be the current trend.

In recent years, breakthroughs in the systematic treatment of liver cancer have been made. TKI drugs such as sorafenib and lenvatinib have been successively approved for first-line treatment of advanced liver cancer (33). Some studies have shown that TACE in combination with TKI therapy improves the prognosis of patients with uHCC compared to TACE monotherapy (34–37). However, the combination of TACE and TKIs as therapy did not achieve a good conversion rate (again, only 11%) (16). ICI therapies, such as programmed cell death protein 1/programmed death-ligand 1 inhibitors, have also shown significant survival benefits in patients with advanced liver cancer and have been approved for second-line treatment (38). Due to the complementary mechanisms of TACE, TKIs, and ICIs, this triple-therapy approach achieved a higher conversion rate. In this analysis, triple therapy achieved a high conversion rate of 42%, which meant that nearly half of patients with unresectable liver cancer underwent surgical removal.

Conversion therapy allows patients with initially uHCC to undergo surgery after successful downstaging. However, this poses the following question: how might one define the criteria for successful downstaging? It is generally accepted that a reduction in tumor size may be a valid evaluation indicator, but estimates based solely on changes in tumor size can be misleading (39). At present, mRECIST is the most commonly used tumor-response assessment. Some researchers have also used ORR criteria for surgical resection after downstaging. However, taking TACE alone as an example, in our analysis, although the ORR of the TACE monotherapy group was as high as 44%, the conversion rate was only 11%. In other words, >30% of patients met the ORR criteria but did not meet the criteria for resection. Overall, a higher ORR did correlate with a higher conversion rate, but ORR cannot be used as the indicator of resection criteria alone. It is important to note that the time between the initiation of treatment and confirmation of downstaging was mostly <6 months, with an average of 2.27 months. During this period, the patient’s resectability should be repeatedly assessed early.

It was unclear and controversial whether further surgery is necessary for patients who have received conversion therapy and subsequently met the criteria for surgery after successful downstaging. Zhang et al. (23) thought that the OS of patients with initially unresectable liver cancer who met the surgical criteria after downstaging but refused surgery was similar to that of patients who received surgical resection after downstaging. On the other hand, Shi et al. (24) suggested that patients who met the surgical resection criteria should undergo surgical resection probably because most patients had residual living tumor cells that would regenerate or metastasize if not surgically removed. As can be seen from our analysis, the postoperative OS of patients who underwent surgical resection after conversion therapy was higher than that of patients who received overall conversion therapy. Therefore, we thought that, for patients with initially uHCC, subsequent surgery after downstaging can improve the survival of patients, thus providing survival benefits. Meanwhile, the incidence of major postoperative complications, such as biliary leakage (7%) and liver failure (3%), in patients with initially uHCC who underwent surgical resection after conversion therapy was similar to that in patients with early liver cancer who underwent initial surgical resection. A high OS and the absence of increased postoperative complication rates suggested that successful downstaging followed by surgical treatment resulted in more favorable outcomes for patients.

The present meta-analysis had several limitations. First, most of the included studies were retrospective small-sample studies. In addition, the inclusion and exclusion criteria for patients in each study were not entirely the same, and there were also subjective judgments of tumor resectability made in the process of conversion therapy related to the preference and experience of each institution. Since no specific details of liver function and tumor stage were provided in each study, there were also no separate detailed subgroups, the effects of liver function and tumor stage on translational therapy for patients with uHCC cannot be made clear.

According to current studies on TACE conversion therapies for uHCC, triple therapy, as a type of conversion therapy of TACE in combination with TKIs and ICIs, has the highest conversion rate. Surgical resection with successful conversion therapy could maximize the outcome of patients with uHCC. Meanwhile, for patients with an early radiation response and reduced or normalized tumor markers, repeated resectable evaluations were required within 6 months (especially within 2 months) after initiation of conversion therapy. However, more prospective randomized controlled studies with larger samples are needed in the future to provide reasonable guidance for conversion therapy.

## Data availability statement

The original contributions presented in the study are included in the article/**Supplementary material**. Further inquiries can be directed to the corresponding author.

## Ethics statement

Ethical review and approval was not required for the study on human participants in accordance with the local legislation and institutional requirements. Written informed consent for participation was not required for this study in accordance with the national legislation and the institutional requirements.

## Author contributions

WL and JL contributed to the conception and design of the study. WL and YP conducted the literature search and extracted the data. ZW was involved in the resolution of all the arguments. WL conducted the data analysis and wrote the manuscript. All authors contributed to the article and approved the submitted version.

## Funding

This work was supported by the Hebei Provincial Key Research Project (21377767D) and the Hebei Provincial Innovation Foundation for Postgraduate (CXZZSS2022137).

## Acknowledgments

We thank LetPub (www.letpub.com) for its linguistic assistance during the preparation of this manuscript.

## Conflict of interest

The authors declare that the research was conducted in the absence of any commercial or financial relationships that could be construed as a potential conflict of interest.

## Publisher’s note

All claims expressed in this article are solely those of the authors and do not necessarily represent those of their affiliated organizations, or those of the publisher, the editors and the reviewers. Any product that may be evaluated in this article, or claim that may be made by its manufacturer, is not guaranteed or endorsed by the publisher.
