# Supplementary material for: Efficacy of transarterial chemoembolization monotherapy or combination conversion therapy in unresectable hepatocellular carcinoma: A systematic review and meta-analysis
Source: Front Oncol. 2022 Aug 1;12:930868. doi: 10.3389/fonc.2022.930868 (PMC9377519; doi:10.3389/fonc.2022.930868)
Supplement: Supplementary file 1 [file DataSheet_1.docx]

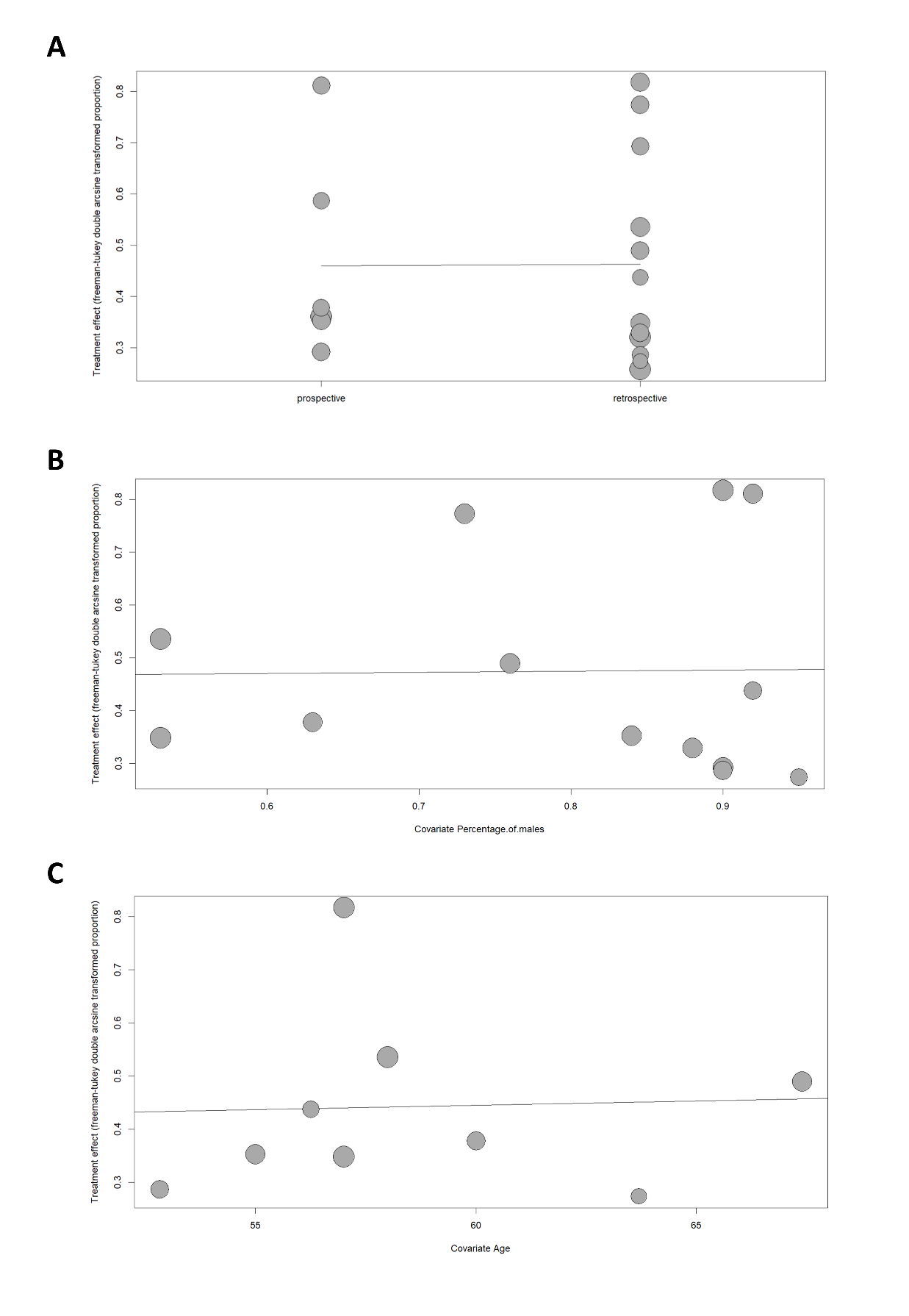


**Supplementary Figure 1:** **Meta-regression of the origin of heterogeneity on conversion rate.**


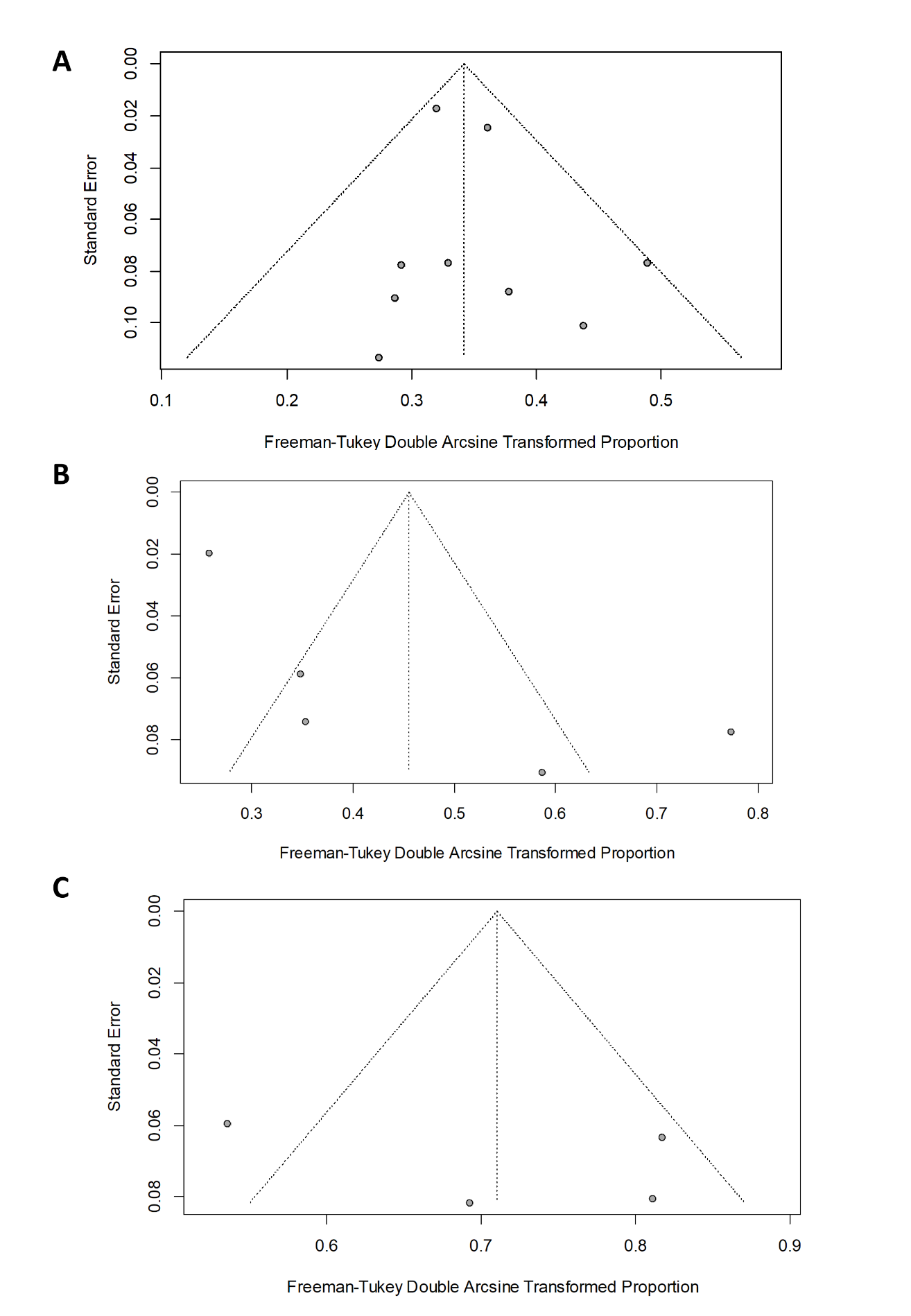


**Supplementary Figure 2: Funnel plots for the assessment of publication bias.**
